# Acute severe carbon monoxide poisoning followed by recurrent cerebral infarction: a case report

**DOI:** 10.3389/fmed.2026.1819385

**Published:** 2026-05-13

**Authors:** Xinyuan Han, Zehua Liu

**Affiliations:** 1 Shaanxi Provincial Rehabilitation Hospital, Xi'an, China; 2 Department of Traditional Chinese Medicine, Shaanxi Provincial People's Hospital, Xi'an, China

**Keywords:** carbon monoxide poisoning, case report, cerebral infarction, child, neuroimaging

## Abstract

**Background:**

Carbon monoxide (CO) poisoning can lead to various neurological complications; however, recurrent cerebral infarction as a clinical manifestation is rarely reported.

**Case presentation:**

We report a case of a previously healthy 13-year-old girl who was admitted to the intensive care unit (ICU) with coma following acute severe CO poisoning (carboxyhemoglobin level 46%). She received multi-organ function monitoring and life support in the ICU. After hyperbaric oxygen therapy, her consciousness initially recovered. However, on day 5 post-intoxication, she acutely developed somnolence and right-sided hemiparesis and was readmitted to the ICU. Neuroimaging revealed acute cerebral infarction in the left basal ganglia region accompanied by severe stenosis of the left M1 segment of the middle cerebral artery (MCA). Although the affected vessel showed recanalization after comprehensive treatment, a follow-up MRI on day 19 unexpectedly identified a new acute infarction in the left cerebral peduncle. A systematic workup revealed no evidence of definite embolic sources. After 8 weeks of comprehensive treatment and rehabilitation, she was discharged with improved motor function (right limb strength graded 4/5 on the Medical Research Council [MRC] scale) but residual anterograde amnesia and personality changes.

**Conclusion:**

This case presents a rare clinical course of recurrent cerebral infarction following acute severe CO poisoning, highlighting that even with early symptomatic improvement, vigilance for delayed cerebrovascular events and long-term neurofunctional and neuropsychological follow-up are essential.

## Introduction

Carbon monoxide (CO) poisoning is a common acute intoxication in clinical practice, leading to diverse and complex neurological impairments (1, 2). Studies suggest that patients with CO poisoning have a significantly increased risk of subsequent ischemic stroke (3, 4). However, clinical presentations characterized by recurrent cerebral infarction within a short period are rarely reported, and the underlying pathophysiological mechanisms remain to be elucidated. We report a case of recurrent cerebral infarction in a child following acute severe CO poisoning, analyzing the clinical course and dynamic imaging evolution to explore possible mechanisms and provide insights for clinical identification and management.

## Case presentation

A previously healthy 13-year-old girl was found unconscious in a confined space (with deceased relatives from a gas leak present on scene) at an unknown specific time and was emergently admitted to the intensive care unit (ICU). The patient’s parents and immediate family have no history of cerebrovascular disease, thrombotic disorders, or inherited hypercoagulable conditions. The patient had no acquired thrombotic risk factors such as prolonged immobilization, obesity, or dehydration, and no prior history of thrombotic events. The patient lives with her parents and has a harmonious family relationship. The accident was caused by a gas leak at home, in which a relative died from poisoning. The patient received psychological support during hospitalization. On admission, vital signs were: body temperature 36.3 °C, pulse 112 beats/min, respiratory rate 13 breaths/min, blood pressure 102/66 mmHg, and finger pulse oximetry 89%. Neurological examination revealed bilaterally equal and round pupils (4 mm) with intact light reflexes, and globally reduced muscle tone in all four limbs. The Glasgow Coma Scale (GCS) score was 8 (eye opening 2, verbal response 2, motor response 4). Arterial blood gas analysis demonstrated hypoxia and metabolic acidosis (pH 7.28, PaO₂ 55 mmHg, lactate 5.2 mmol/L). The carboxyhemoglobin (COHb) level was markedly elevated at 46%. Non-contrast head CT and electrocardiogram showed no acute abnormalities.

The patient was diagnosed with acute severe CO poisoning. She received multi-organ function monitoring and life support in the ICU, along with hyperbaric oxygen therapy (2.0 atmospheres absolute every 12 h). 24 h after treatment, her consciousness gradually cleared and she could engage in simple communication. During days 2–4, neurological function continuously improved; she was able to ambulate slowly with assistance but exhibited significant anterograde amnesia. After clinical stabilization, she was transferred from the ICU to the general ward.

**Figure 1:**
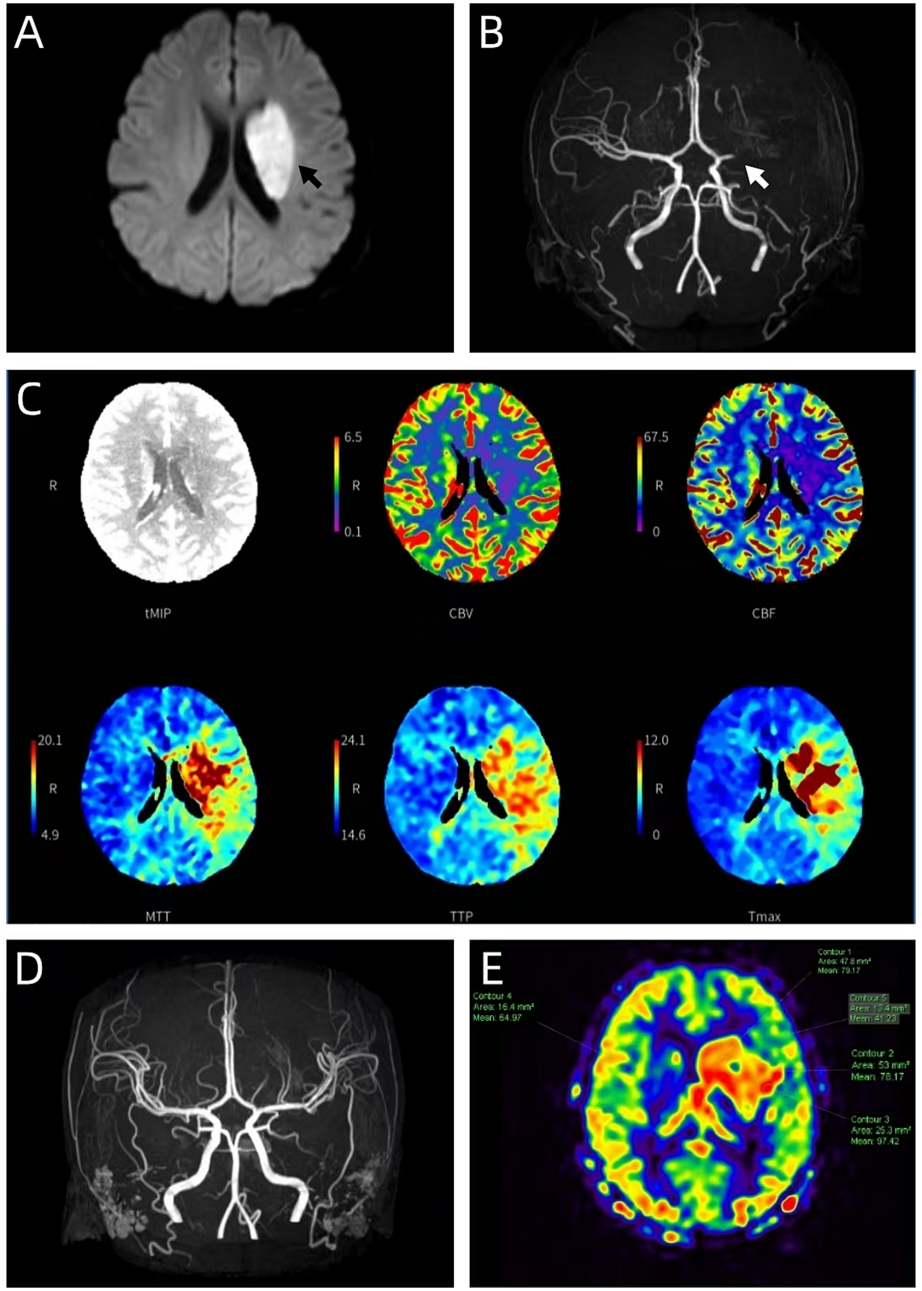
Neuroimaging findings during the initial acute phase and following treatment. **(A)** Brain DWI sequence shows a patchy area of hyperintensity in the left basal ganglia and periventricular region, consistent with acute cerebral infarction (arrow). **(B)** Brain MRA reveals focal luminal narrowing and significant stenosis of the left M1 segment of the middle cerebral artery (arrow), with poor visualization of the distal segment. **(C)** Brain CT perfusion (CTP) imaging demonstrates a large area of hypoperfusion within the left MCA territory (involving the frontal, parietal, and temporal lobes, basal ganglia, and insula). Quantitative analysis indicates a core infarct volume of 7.9 mL, a hypoperfusion volume of 66.5 mL, and a significant ischemic penumbra (mismatch volume 58.6 mL, mismatch ratio 8.4). **(D)** Follow-up MRA after treatment shows restoration of the lumen in the left M1 segment, with a normal caliber and course, and no significant stenosis. This dynamic evolution from stenosis to normality is consistent with recanalization following resolved cerebral vasospasm. **(E)** Repeat perfusion imaging shows significantly increased perfusion (hyperperfusion) around the original left basal ganglia infarction, consistent with post-ischemic hyperperfusion.

On day 5 post-intoxication, the patient’s condition took a turn, presenting with somnolence and right-sided hemiparesis. Due to clinical deterioration, she was readmitted to the ICU. Neurological examination revealed a GCS score of 13 (somnolence), right central facial palsy, muscle strength graded 1/5 in the right upper limb and 2/5 in the right lower limb (Medical Research Council [MRC] scale), and a right Babinski sign. Based on these focal neurological deficits, the National Institutes of Health Stroke Scale (NIHSS) score was 17 (level of consciousness 1 point, questions 2 points, commands 2 points, facial palsy 2 points, right arm motor 4 points, right leg motor 3 points, sensory 1 point, language 1 point, dysarthria 1 point), and the modified Rankin Scale (mRS) score was 4. Brain MRI with diffusion-weighted imaging (DWI) demonstrated acute cerebral infarction in the left basal ganglia and periventricular region. Magnetic resonance angiography (MRA) revealed severe stenosis of the left M1 segment of the middle cerebral artery (MCA), with a core infarct volume of 7.9 mL (Figures 1A–C). After evaluation, the time from symptom onset exceeded the standard window for intravenous thrombolysis (>4.5 h). MRA showed severe stenosis of the left M1 segment of the middle cerebral artery rather than complete occlusion. Imaging could not clearly differentiate between vasospasm and thrombosis. Therefore, the patient did not meet typical criteria for endovascular thrombectomy. The risks and benefits of reperfusion therapy were unclear. Thus, no reperfusion therapy was performed. On the basis of ongoing hyperbaric oxygen and rehabilitation therapy, the patient was started on aspirin (100 mg once daily) for antiplatelet effect and atorvastatin (20 mg once daily) for endothelial stabilization. Given the absence of atherosclerotic risk factors, cerebral vasospasm was highly suspected, and empirical continuous intravenous infusion of nimodipine was initiated for anti-vasospastic therapy (starting at 5 mL/h, gradually titrated to 10 mL/h based on blood pressure tolerance). After 7 days of treatment, follow-up MRA showed complete recanalization of the left MCA (Figure 1D), and perfusion imaging revealed improved perfusion in the peri-infarct region (Figure 1E). This dynamic evolution suggested that the initial stenosis might be related to cerebral vasospasm or spontaneous thrombolysis.

**Figure 2:**
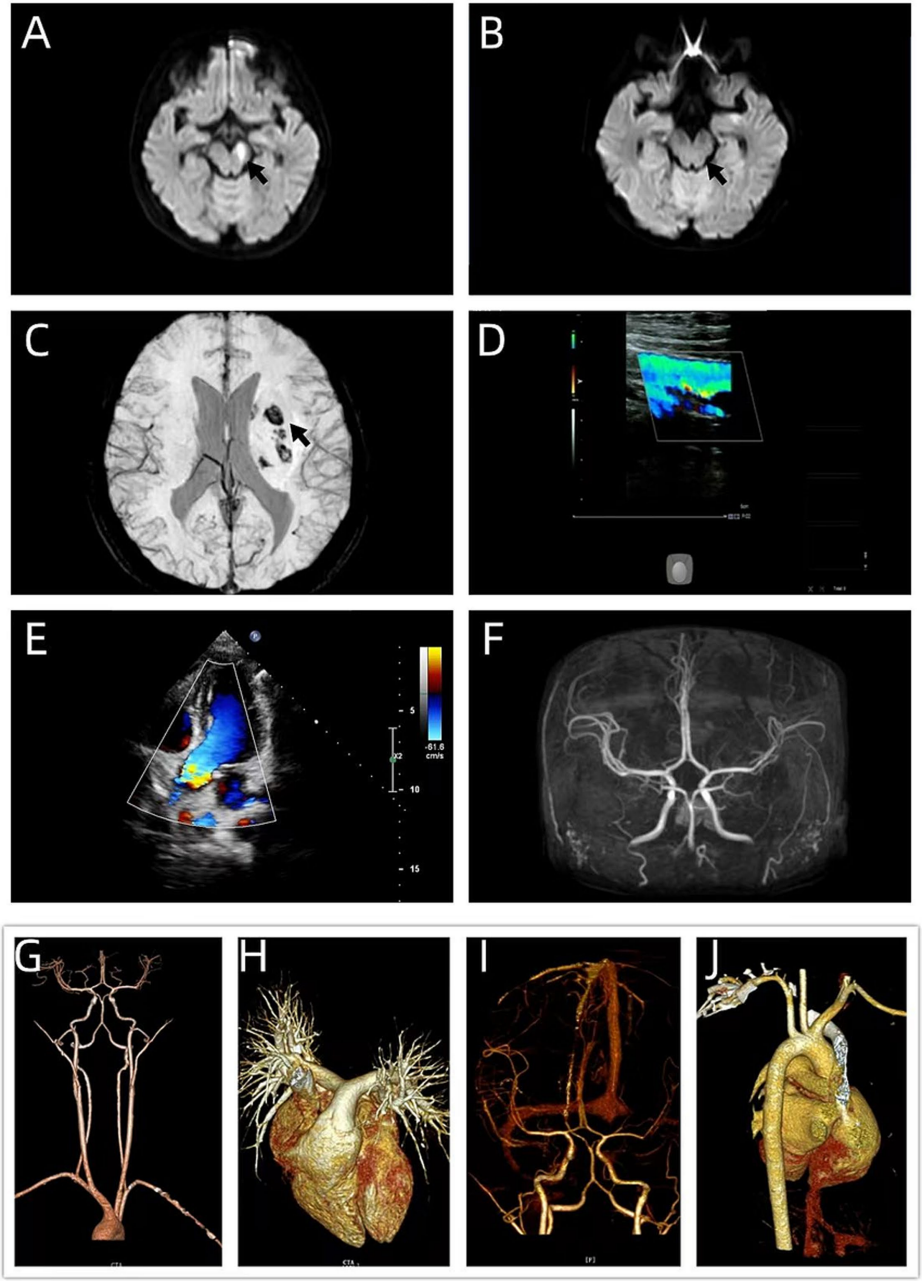
Follow-up imaging and systemic evaluation for embolism. **(A,B)** Comparison of brain DWI sequences. **(A)** (Current study) shows a new patchy hyperintensity in the left cerebral peduncle, consistent with acute cerebral infarction (arrow). **(B)** (Study from 14 days prior) shows no abnormal signal in the corresponding region (arrow). **(C)** Follow-up DWI and susceptibility-weighted imaging (SWI) reveals decreased DWI signal in the original left basal ganglia-periventricular infarction. SWI shows punctate hypointense foci within the lesion (arrow), consistent with subacute infarction accompanied by hemorrhagic transformation. **(D,E)** Embolism workup. **(D)** (Color Doppler ultrasound of the extremities) and **(E)** (Contrast transthoracic echocardiography) show no definitive thrombotic source or right-to-left shunt (RLS grade 0). **(F–J)** Systemic vascular imaging evaluation. Brain MRA, neck CTA, pulmonary artery CTA, pulmonary vein and left atrium CTA, intracranial CTA, and ascending aorta CTA show no evidence of arterial stenosis, dissection, or embolism.

On day 19 post-intoxication, although the patient had no new focal neurological deficits, routine follow-up MRI revealed a new acute infarction in the left cerebral peduncle (Figures 2A–C). The DWI sequence showed markedly high signal intensity in this lesion, while the corresponding region on MRI 14 days earlier was completely normal (Figure 2B), consistent with features of a fresh infarction. This allows differentiation from the subacute evolution of the original left basal ganglia infarction. Systemic imaging studies (Figures 2D–J) and hematological screening, including coagulation function, D-dimer, antiphospholipid antibodies, protein C, protein S, antithrombin, lupus anticoagulant, homocysteine, antinuclear antibody profile, and anti-neutrophil cytoplasmic antibodies, all of which were within normal limits, revealed no evidence of embolism. Considering the history of CO poisoning and the transient occlusion-recanalization process of the MCA, the risk of persistent vasospasm was suspected. Therefore, nimodipine was switched to an oral regimen of 60 mg every 4 h to intensify anti-vasospastic therapy, while aspirin and atorvastatin were continued to address thrombotic risk.

Thereafter, the patient continued to receive the aforementioned comprehensive treatment, and her condition gradually stabilized. On day 20, her motor function further improved, and she was transferred from the ICU to the general ward for continued care. After 8 weeks of comprehensive treatment, the patient was discharged in stable condition (Table 1). At discharge, she was alert with fluent speech; right limb muscle strength had improved to grade 4/5. However, she exhibited residual anterograde amnesia and personality changes, including pressured speech and emotional lability. The final diagnoses were: acute severe carbon monoxide poisoning; recurrent cerebral infarction (left basal ganglia-periventricular region, left cerebral peduncle); and post-intoxication cognitive dysfunction and behavioral changes. Discharge medications included aspirin (100 mg once daily) and atorvastatin (20 mg once daily) for secondary cerebrovascular prevention. Based on the occurrence of two cerebral infarctions, with the second occurring during treatment—highly suggestive of persistent vasospasm risk—oral nimodipine (60 mg every 4 h) was continued for anti-vasospastic therapy. This regimen followed the preventive protocol for vasospasm after subarachnoid hemorrhage and represented off-label use, requiring regular outpatient blood pressure monitoring and dose adjustment. After discharge, the patient continued outpatient neuropsychological rehabilitation. Six-month follow-up showed that the patient’s right limb muscle strength had recovered to 4+/5, and she could walk independently without instability. Recent memory had significantly improved, allowing her to complete daily learning tasks. The symptoms of pressured speech and emotional lability had diminished, and her communication with classmates was normal. She had successfully returned to school, performed well academically, and required no special learning support.

**Table 1:** Timeline of clinical events, imaging findings, management decisions, and outcomes.

Time point	Neurological events	Imaging findings	Management decisions	Clinical outcomes
Admission	Coma (GCS 8)	Non-contrast head CT: no acute abnormality	ICU admission, multi-organ support, hyperbaric oxygen therapy	—
Days 2–4	Consciousness recovery, anterograde amnesia	—	Transferred to general ward	Improved
Day 5	Somnolence, right hemiparesis (NIHSS 17)	MRI: acute left basal ganglia infarction; MRA: severe left M1 stenosis	ICU readmission, aspirin, atorvastatin, intravenous nimodipine (anti-vasospastic therapy)	—
Day 12	No improvement in hemiparesis	MRA: complete recanalization of left MCA; perfusion imaging: improved peri-infarct perfusion	Continued anti-vasospastic therapy	—
Day 19	Asymptomatic	MRI: new acute infarction in left cerebral peduncle	Nimodipine switched to oral 60 mg every 4 h to intensify anti-vasospastic therapy; continued aspirin and atorvastatin	New infarction detected (asymptomatic)
Day 20 to discharge	Improved motor function	—	Rehabilitation, discharge preparation	Transferred from ICU to general ward
6-month follow-up	Improved cognition and behavior	—	All medications discontinued	Returned to school

This timeline summarizes the key clinical events, imaging findings, management decisions, and outcomes during hospitalization and follow-up. Anti-vasospastic therapy with nimodipine was based on imaging features suggestive of reversible cerebral vasospasm, following the standard protocol for subarachnoid hemorrhage (off-label use).

## Discussion

This case describes recurrent cerebral infarction in a child following acute severe CO poisoning. The clinical course was characterized by an initial infarction in the left basal ganglia on day 5 post-intoxication, which recanalized after treatment, followed by a new infarction in the left cerebral peduncle detected on day 19.

The established mechanisms of CO-induced brain injury include hypoxia, inflammatory activation, and mitochondrial dysfunction (5–8). In addition, CO can affect platelet function, activate neutrophils, and trigger inflammatory cascades (9, 10). This inflammatory response can persist long after the acute exposure and does not correlate directly with carboxyhemoglobin levels (10, 11). These pathological processes may converge on the cerebral vasculature, leading to cerebral vasospasm and local thrombosis. Hypercoagulability (3), endothelial injury (6), and delayed inflammation (10) may all contribute to thrombosis, but none alone can fully explain the reversible “stenosis-recanalization” dynamic evolution observed on MRA—a phenomenon that could be attributed to either resolution of vasospasm or spontaneous thrombolysis, or it might suggest another transient arteriopathic process. In summary, this case likely involves the interplay of multiple mechanisms: CO poisoning creates a vascular microenvironment conducive to both vasospasm and thrombosis. The “stenosis-recanalization” dynamic evolution seen on MRA is consistent with the features of reversible cerebral vasospasm, but it does not allow for a definitive distinction between vasospasm and spontaneous thrombolysis. Atorvastatin was used in this non-atherosclerotic pediatric patient primarily for its pleiotropic effects (anti-inflammatory and endothelial stabilization), rather than for lipid-lowering purposes.

The use of nimodipine in this case was primarily based on the following considerations: the patient had no atherosclerotic risk factors, and the dynamic “stenosis-recanalization” evolution on MRA suggested the possibility of reversible cerebral vasospasm. There is currently no standardized treatment protocol for vasospasm following CO poisoning. Therefore, we referred to the nimodipine regimen used for vasospasm after subarachnoid hemorrhage (intravenous infusion starting at 5 mL/h and titrated to 10 mL/h, followed by oral administration of 60 mg every 4 h). Blood pressure remained stable during intravenous infusion (systolic blood pressure >90 mmHg), and the risk of hypotension was managed through close monitoring. The clinical outcomes—vessel recanalization and the absence of new events at 6 months—suggest that anti-vasospastic therapy may have been beneficial.

The causes of childhood stroke are diverse, including developmental vasculopathy, arterial dissection, thrombosis, and inflammatory arteriopathy (12). Carbon monoxide poisoning itself can increase the risk of cerebrovascular events (3) and worsen brain injury through neurotoxic mechanisms (6). In this case, differential diagnosis should consider the following childhood stroke etiologies: developmental vasculopathy (e.g., moyamoya disease)—not supported, as CTA and MRA showed no abnormalities (13); occult arterial dissection—cannot be completely excluded, as high-resolution vessel wall imaging was not performed (14); transient in-situ thrombosis—could explain the “stenosis-recanalization” phenomenon and involvement of both anterior and posterior circulations; delayed inflammatory arteriopathy—the time window is compatible, but there is no imaging evidence of vasculitis (15). The second infarction (left cerebral peduncle, posterior circulation) cannot be explained by an isolated left M1 lesion alone, suggesting more widespread vascular involvement rather than a single focal event. Typical delayed encephalopathy is mainly characterized by cognitive impairment and confusion. In contrast, this case is defined by recurrent and multifocal cerebral infarction. Imaging showed dynamic “stenosis-recanalization” changes. This suggests that the underlying mechanism may primarily involve vasospasm and thrombosis. The developing cerebral vasculature in children may exhibit heightened susceptibility to inflammatory mediators and oxidative stress induced by CO poisoning, potentially explaining the occurrence of vasospasm and local thrombosis in this pediatric patient (9, 11). The imaging findings differed from the classic globus pallidus and white matter lesions characteristic of delayed encephalopathy (16–18). This distinction highlights the heterogeneity of CO-related neurological complications, suggesting that the stroke-predominant phenotype may arise from mechanisms independent of those underlying traditional delayed encephalopathy.

## Conclusion

The dynamic imaging evolution in this case suggests that vasospasm and thrombosis may both contribute to the disease course, although the exact mechanism remains unclear. Even with early symptomatic improvement, vigilance for delayed cerebrovascular events and long-term imaging and neuropsychological follow-up are necessary.

### Limitations

As a single clinical observation, the generalizability of our findings is inherently limited. Direct dynamic imaging evidence of cerebral vasospasm and pathological confirmation of thrombosis are lacking. MRA was performed only at specific time points, potentially missing transient stenosis-recanalization events in other vascular territories. High-resolution vessel wall imaging was not performed in this case. Therefore, occult arterial dissection, focal vasculitis, or subtle vessel wall pathology cannot be excluded. This is an important limitation. In addition, prolonged cardiac monitoring (>72 h) was not performed, so paroxysmal atrial fibrillation or other cardioembolic sources cannot be completely excluded. Furthermore, the follow-up period was relatively short, and long-term prognosis as well as neuropsychiatric outcomes require continued assessment.

#### Patient perspective

On behalf of the patient and her family, we express our sincere gratitude to the medical team for their dedicated care. This experience has profoundly impressed upon us the severity of carbon monoxide poisoning and the critical importance of long-term rehabilitation. We hope that publication of this case will raise public awareness of gas safety and offer encouragement to other families facing similar challenges.

## Data Availability

The original contributions presented in the study are included in the article/supplementary material, further inquiries can be directed to the corresponding author.

## References

[1] Jang DH Piel S Greenwood JC Ehinger JK Kilbaugh TJ. Emerging cellular-based therapies in carbon monoxide poisoning. Am J Physiol Cell Physiol. (2021) 321:C269–75. doi: 10.1152/ajpcell.00022.2021, 34133239 PMC8424679

[2] Dent MR Rose JJ Tejero J. Carbon monoxide poisoning: from microbes to therapeutics. Annu Rev Med. (2024) 75:337–51. doi: 10.1146/annurev-med-052422-020045, 37582490 PMC11160397

[3] Lin CW Chen WK Hung DZ Chen YW Lin CL Sung FC . Association between ischemic stroke and carbon monoxide poisoning: a population-based retrospective cohort analysis. Eur J Intern Med. (2016) 29:65–70. doi: 10.1016/j.ejim.2015.11.025, 26703428

[4] Mo J Li Z Lin Z Liu P Xu W Huang Z . A woman with carotid atherosclerotic plaques suffered a massive cerebral infarction after carbon monoxide poisoning: a case report. Heliyon. (2024) 10:e39896. doi: 10.1016/j.heliyon.2024.e39896, 39524876 PMC11550115

[5] Weaver LK. Clinical practice: carbon monoxide poisoning. N Engl J Med. (2009) 360:1217–25. doi: 10.1056/NEJMcp0808891 19297574

[6] Rose JJ Wang L Xu Q McTiernan CF Shiva S Tejero J . Carbon monoxide poisoning: pathogenesis, management, and future directions of therapy. Am J Respir Crit Care Med. (2017) 195:596–606. doi: 10.1164/rccm.201606-1275CI, 27753502 PMC5363978

[7] Wang T Zhang Y. Mechanisms and therapeutic targets of carbon monoxide poisoning: a focus on reactive oxygen species. Chem Biol Interact. (2024) 403:111223. doi: 10.1016/j.cbi.2024.111223, 39237073

[8] Bungatavula D Greenwood JC Shofer FS Buehler G Kao SH Kelly M . Blood cells as a cellular biomarker for mitochondrial function in an experimental model of acute carbon monoxide poisoning with treatment. J Med Toxicol. (2025) 21:327–35. doi: 10.1007/s13181-025-01077-6, 40295447 PMC12204972

[9] Arya AK Sethuraman K Waddell J Cha YS Liang Y Bhopale VM . Inflammatory responses to acute carbon monoxide poisoning and the role of plasma gelsolin. Sci Adv. (2025) 11:eado9751. doi: 10.1126/sciadv.ado9751, 39919185 PMC11804920

[10] Thom SR Bhopale VM Han ST Clark JM Hardy KR. Intravascular neutrophil activation due to carbon monoxide poisoning. Am J Respir Crit Care Med. (2006) 174:1239–48. doi: 10.1164/rccm.200604-557OC, 16931637 PMC2648106

[11] Kuroda H Fujihara K Kushimoto S Aoki M. Novel clinical grading of delayed neurologic sequelae after carbon monoxide poisoning and factors associated with outcome. Neurotoxicology. (2015) 48:35–43. doi: 10.1016/j.neuro.2015.03.002, 25757834

[12] Sutherly LJ Malloy R. Risk factors of pediatric stroke. J Neurosci Nurs. (2020) 52:58–60. doi: 10.1097/JNN.0000000000000489 31985549

[13] Pangprasertkul S Borisoot W Buawangpong N Sirikul W Wiwattanadittakul N Katanyuwong K . Comparison of arterial ischemic and hemorrhagic pediatric stroke in etiology, risk factors, clinical manifestations, and prognosis. Pediatr Emerg Care. (2022) 38:e1569–73. doi: 10.1097/PEC.0000000000002614, 35113509

[14] Mattay RR Saucedo JF Lehman VT Xiao J Obusez EC Raymond SB . Current clinical applications of intracranial Vessel Wall MR imaging. Semin Ultrasound CT MR. (2021) 42:463–73. doi: 10.1053/j.sult.2021.07.004 34537115 PMC8453001

[15] Cao Y Sun Y Yi Z Meng W Zhao X Feng X . Assessment of central nervous system vasculitis in children based on high-resolution vascular wall imaging. Rheumatol Adv Pract. (2024) 8:rkae038. doi: 10.1093/rap/rkae038 38605731 PMC11009033

[16] Jiang W Zhao Z Wu Q Wang L Zhou L Li D . Study on brain structure network of patients with delayed encephalopathy after carbon monoxide poisoning: based on diffusion tensor imaging. Radiol Med. (2021) 126:133–41. doi: 10.1007/s11547-020-01222-x, 32557108

[17] Ma Y Lv W Hu H Ma Y Ma Y. Risk factors and outcome analysis of delayed neurological sequelae in elderly patients with carbon monoxide poisoning. Undersea Hyperb Med. (2025) 52:283–92. doi: 10.22462/700, 41223390

[18] Gao Y Gu H Yang J Yang L Li Z Zhang J. Prognosis of patients in prolonged coma after severe carbon monoxide poisoning. Hum Exp Toxicol. (2021) 40:1355–61. doi: 10.1177/0960327121997992, 33641437

